# Metatranscriptomic Profiling Reveals the Effect of Breed on Active Rumen Eukaryotic Composition in Beef Cattle With Varied Feed Efficiency

**DOI:** 10.3389/fmicb.2020.00367

**Published:** 2020-03-13

**Authors:** Yawei Zhang, Fuyong Li, Yanhong Chen, Hao Wu, Qingxiang Meng, Le Luo Guan

**Affiliations:** ^1^State Key Laboratory of Animal Nutrition, College of Animal Science and Technology, China Agricultural University, Beijing, China; ^2^Department of Agricultural, Food and Nutritional Science, University of Alberta, Edmonton, AB, Canada

**Keywords:** beef cattle, breed, feed efficiency, eukaryotes, fungi, protozoa, metatranscriptomics

## Abstract

Exploring the compositional characteristics of rumen eukaryotic community can expand our understanding of their role in rumen function and feed efficiency. In this study, we applied metatranscriptomics to characterize the active rumen eukaryotic community (protozoa and fungi) in beef cattle (*n* = 48) of three breeds [Angus (AN), Charolais (CH), and Kinsella Composite (KC)] and with divergent residual feed intake (RFI). The composition of active rumen eukaryotic microbiota was evaluated based on enriched 18S rRNAs from the metatranscriptomic datasets. At the phylum level, a total of four protozoal taxa (*Ciliophora*, *Parabasalia*, unclassified *SAR*, and unclassified *Alveolata*), six fungal taxa (*Neocallimastigomycota*, *Basidiomycota*, unclassified *Fungi*, *Mucoromycota*, *Ascomycota*, and *Chytridiomycota*), and one sister group of kingdom *Fungi* (unclassified *Opisthokonta*) were detected with relative abundances higher than 0.01% and in at least 50% of animals within each breed. Among these, *Ciliophora*, *Parabasalia*, unclassified *Opisthokonta*, and *Neocallimastigomycota* were the top four active eukaryotic phyla. At the genus level, a total of 8 ciliated protozoa, 5 flagellated protozoa, 5 anaerobic fungi, and 10 aerobic fungi taxa were detected, with unclassified *Trichostomatia*, *Tetratrichomonas*, unclassified *Neocallimastigaceae*, and *Pleurotus* being the most predominant taxa of ciliated protozoa, flagellated protozoa, anaerobic fungi, and aerobic fungi, respectively. Differential abundance analysis revealed that breed had a significant effect on the phylogenetic lineages of rumen eukaryotes, and seven fungal taxa were more abundant (linear discriminant analysis score > 2 with *P* < 0.05) in the rumen of KC steers than in the rumen of AN and CH steers. Although principal coordinate analysis (PCoA) revealed that the ruminal active eukaryotic profiles were not distinguishable between high- and low-RFI groups, the diversity indices, including Faith’s phylogenetic diversity (PD), observed operational taxonomic units (OTUs), and Shannon index of rumen eukaryotes were higher in low-RFI steers than those in high-RFI steers. Meanwhile, the abundance of genus *Entodinium* and the kingdom *Fungi* was higher in low-RFI steers than that in high-RFI steers. This information on active rumen eukaryotic microbiota and identified differential abundance of taxa between high- and low-RFI animals suggests the possibility of improving feed efficiency through altering rumen eukaryotic microbiota.

## Introduction

Ruminant production, especially beef cattle operation, is important for the production of high-quality animal protein (meat). Feed efficiency is one of the most important traits in beef cattle production because the feed cost accounts for approximately 60–70% of the total production cost (Karisa et al., 2014). Therefore, improving feed efficiency in beef cattle can maximize the production efficiency when lower or the same amount of feed is consumed by cattle under current management conditions. In addition, cattle with a high feed efficiency emit lesser enteric methane and excrete lesser feces than cattle with a low feed efficiency (Nkrumah et al., 2006; Hegarty et al., 2007). Therefore, there is an urgent need to develop strategies to improve feed efficiency for enhanced economic and environmental sustainability of beef production.

Many factors can affect feed efficiency, including animal genetics, nutrition, biology, physiology, metabolism, behavior, etc. (Kenny et al., 2018). Although it is not well defined, the digestive activity of animals likely contributes to about 10% of the variation in feed efficiency (Richardson and Herd, 2004). Indeed, recently, studies have shown that rumen function (microbial fermentation and nutrient absorption) can impact residual feed intake (RFI), a measure of feed efficiency (Guan et al., 2008; Kong et al., 2016). Ruminants rely on their symbiotic rumen microorganisms to hydrolyze the plant fiber and generate energy and other nutrients (Puniya et al., 2015). The rumen commensal microbiota consists of bacteria, archaea, protozoa, fungi, and phages (Huws et al., 2018). Within the microbial consortium, the eukaryotic community (protozoa and fungi) has been shown to constitute approximately half of the total microbial biomass (Puniya et al., 2015) and is believed to play a critical role in the degradation of lignocellulosic components of the feed particles (Gruninger et al., 2014; Newbold et al., 2015). To date, many studies have reported significant differences in the relative abundance of several rumen bacterial and archaeal phylotypes between efficient and inefficient cattle (Jewell et al., 2015; Myer et al., 2015; Li et al., 2019a). In addition, it has been observed that cattle with a lower feed efficiency possess more complex and diverse rumen bacterial and archaeal communities than cattle with a high feed efficiency (Zhou et al., 2009; Shabat et al., 2016), thereby suggesting that rumen microbiota may play a role in contributing to cattle feed efficiency. However, most of these studies only focused on the prokaryotic community (bacterial and archaeal) and/or assessed the eukaryotic community at the DNA (genomic) level. To date, our knowledge on the ruminal eukaryotic profile is still limited, especially with respect to function and activity, and the linkage between active rumen eukaryotic community and feed efficiency has not yet been reported.

On the other hand, although previous studies have shown that rumen microbial taxonomic profiles are distinguishable among hosts with different genetic backgrounds (Hernandez-Sanabria et al., 2013; Henderson et al., 2015; Li et al., 2019b), only bacteria and archaea were profiled reported in these studies. Therefore, in the present study, we profiled active rumen eukaryotic communities using the metatranscriptomic data generated from 48 beef steers with divergent RFI from three breeds to identify the active protozoal and fungal communities in the rumen and assess whether they are affected by RFI and breed. The objectives of this study were to holistically explore the active rumen eukaryotic profile in beef cattle and to investigate its relationship with cattle RFI as well as host breed.

## Materials and Methods

### Metatranscriptomic Data

RNA-seq data were obtained from our previously published study (Li et al., 2019a), under accession number PRJNA448333, for the sequences deposited in the Sequence Read Archive of the National Center for Biotechnology Information. These data were generated by extracting total RNA extracted from rumen content samples collected from 48 steers (selected from a herd of 738 beef cattle) from three breeds [two purebreds, Angus (AN) and Charolais (CH), and one crossbred, Kinsella Composite (KC)], with eight animals per RFI category per breed (a total of 16 per breed) who were raised under the same feedlot conditions. Briefly, these cattle were fed with the same finishing diet, consisting of 80% barley grain, 15% barley silage, and 5% rumensin pellet supplement (Killam Tag 849053; Hi-Pro Feeds, Westlock, AB, Canada). The daily dry matter intake (DMI) and average daily gain (ADG) were measured over a test period of 70–73 days using GrowSafe System (GrowSafe Systems Ltd., Airdrie, AB, Canada), and RFI was calculated as described by Basarab et al. (2011). Steers were slaughtered before feeding, and the rumen content samples were collected within half an hour after slaughter. In total, 3,087.41 M paired-end reads [64.32 ± 0.74 M (mean ± SEM) per sample] were used for analysis of active rumen eukaryotic microbiota.

### Assessment of the Active Rumen Eukaryotic Microbiota Using Metatranscriptomics

The RNA-seq dataset was preliminarily processed as described by Li et al. (2016), and reads after quality control and filtration were used in this study. The 18S rRNA reads were extracted by mapping the filtered reads to the rRNA reference database SILVA_SSU (release 119) using SortMeRNA (version1.9) (Kopylova et al., 2012) and subjected to downstream analysis using Quantitative Insights Into Microbial Ecology (QIIME 2, version 2019.4) (Bolyen et al., 2019). The enriched 18S rRNA paired reads were quality-filtered, merged, dereplicated, and chimera-filtered using the q2-dada2 plugin (Callahan et al., 2016) to obtain representative sequence variants (RSVs) and their frequency distribution tables. This process was achieved without trimming or truncating any base at the beginning or end of the sequences, respectively. The RSVs were then used to generate compositional profiles of the active rumen eukaryotic communities. Eukaryotic taxonomic classification was performed using the classify-sklearn command (Pedregosa et al., 2011) of the q2-feature-classifier plugin with a pretrained naive Bayesian classifier. The classifier was pretrained on the Silva 18S rRNA database (release 132) using the fit-classifier-Naive–Bayes method from the q2-feature-classifier plugin. Next, to analyze phylogenetic diversity (PD), a phylogenetic tree was generated using the align-to-tree-mafft-fasttree pipeline integrated in the q2-phylogeny plugin. To assess if sample richness has been fully observed, alpha rarefaction plots were generated based on the Shannon index and Faith’s PD metrics using the alpha-rarefaction visualizer of the q2-diversity plugin. To comparably analyze the eukaryotic diversity among samples, the sequence count of all samples was standardized by rarefying them to the same number of sequences (the smallest sampling size) using the rarefy command of the q2-feature-table plugin. The rarefied feature table and the phylogenetic tree were then used to compute alpha diversity indices, including the Shannon index, observed operational taxonomic units (OTUs), Faith’s PD, and Pielou’s evenness and beta diversity metrics, including unweighted and weighted UniFrac distance, using the core-metrics-phylogenetic pipeline integrated in the q2-diversity plugin.

### Statistical Analysis

In the present study, microbial taxa with a relative abundance higher than 0.01% in at least one animal were considered as being identified, while microbial taxa with a relative abundance higher than 0.01% and presented in at least 50% of animals within each breed were considered as being detected and used for downstream statistical analysis. Eukaryotic composition profiles were summarized at phylum and genus levels, respectively. Relative abundances of microbial taxa were arcsine square root transformed and then compared among different breeds and RFI categories (differential abundance analysis) using the mixed procedure in SAS (version 9.4; SAS Institute Inc., Cary, NC, United States). A mixed effect model was used as follows: *y*_*ij*_ = μ + α*_*i*_* + β*_*j*_* + (α × β)*_*ij*_* + ε*_*ij*_*, where μ is the intercept and ε*_*ij*_* is the residual error term; α*_*i*_*, β*_*j*_*, and (α × β)*_*ij*_* are the fixed effects of *i*th beef breed (AN, CH, and KC), *j*th RFI group (high- and low-RFI), and their interaction, respectively. The significance level was defined as *P* < 0.05. The more stringent linear discriminant analysis (LDA) effect size (LEfSe) was performed to further identify differentially abundant eukaryotic taxa, as described by Segata et al. (2011), and taxa with LDA score > 2 and *P* < 0.05 were considered to be significantly different. The difference in alpha diversity indices among the three breeds and between different RFI categories and their interaction was tested with the same mixed effect model using SAS. PCoA was used to visualize rumen eukaryotic communities based on the unweighted and weighted UniFrac distances at the RSVs level. Permutational multivariate analysis variance (PERMANOVA) was used to test the dissimilarity of active rumen eukaryotic profiles among the different breeds and RFI categories using the beta-group-significance command of the q2-diversity plugin (Anderson, 2001).

## Results and Discussion

### Rumen Eukaryotic Communities Identified by Enriched 18S rRNA Sequences

From the 3,087.41 M metatranscriptomic dataset, a total of 511.85 M paired-end reads belonging to the 18S rRNA gene (10.66 ± 0.74 M) (16.55%) were enriched. The average number of 18S rRNA was found to be over 10-fold higher than that in a recent rumen metatranscriptomic study (945,524 reads) on dairy cow (Comtet-Marre et al., 2017). After quality control, combining paired-end reads, and filtering chimeras, a total of 149,672,807 high-quality 18S rRNA sequences (3,118,183 ± 209,081) were generated. These sequences were further categorized as 38,064 RSVs of 18S rRNA, with a length of 129.79 ± 22.5 bases. The Good’s coverage was >99.99 (±0.0006%), suggesting that the sequencing depth of our metatranscriptome had sufficient coverage for the eukaryotic communities.

**FIGURE 1:**
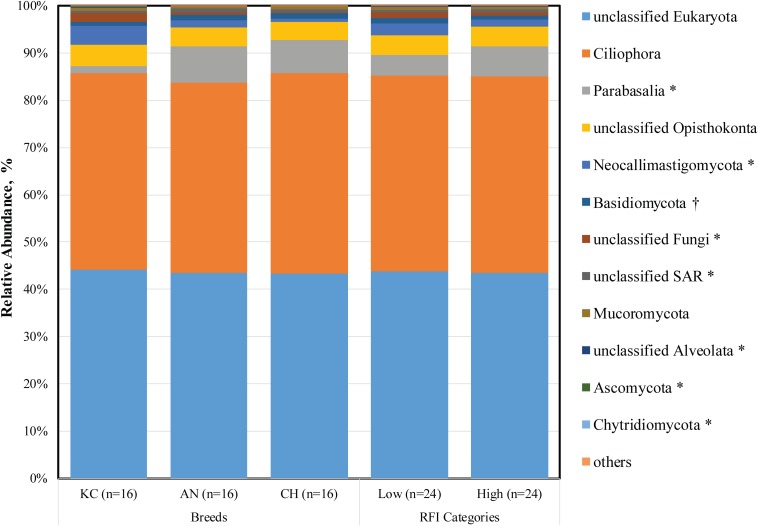
Active rumen eukaryotic composition at the phylum level in beef cattle of three breeds and with divergent residual feed intake (RFI). Taxa name with asterisk (*) represents significant breed effect (*P* < 0.05), and taxa name with dagger (†) represents significant RFI effect (*P* < 0.05).

Taxonomic profiling revealed a total of 15 eukaryotic taxa at the phylum level as being identified, 12 of which were classified as being detected (Figure 1) according to the cutoff defined in the section “Materials and Methods.” Each detected eukaryotic taxon was found in at least 45 out of 48 samples (>93.75%), confirming the presence of a core active rumen eukaryotic community, as reported previously for bacteria, archaea, and protozoa at the DNA level (Henderson et al., 2015). Notably, 43.62 ± 0.15% of sequences were assigned to unclassified eukaryotes (Figure 1 and Supplementary Table S1), indicating that knowledge on taxonomic identification of rumen eukaryotes is still very limited. However, to better represent the active rumen eukaryotic communities and to reveal the potential associations between microbial taxa and host phenotype, all detected taxa, including the unnamed and/or unclassified taxa were included in the analysis.

Among the rest of 11 detectable active eukaryotic taxa, the *Ciliophora* (41.47 ± 1.43%), unclassified *SAR* (0.66 ± 0.04%), unclassified *Alveolata* (0.14 ± 0.02%), and *Parabasalia* (5.36 ± 1.35%) were categorized as protozoa (Supplementary Table S1), including ciliated (*Ciliophora*) and flagellated (*Parabasalia*) protozoa and their sister groups (Warren and Esteban, 2019). In addition, six taxa belonging to the kingdom *Fungi* were detected (Supplementary Table S1), including *Neocallimastigomycota* (2.00 ± 0.44%), *Basidiomycota* (1.07 ± 0.09%), unclassified *Fungi* (0.88 ± 0.20%), *Mucoromycota* (0.55 ± 0.03%), *Ascomycota* (0.09 ± 0.05%), and *Chytridiomycota* (0.008 ± 0.001%), with *Neocallimastigomycota* representing the rumen-specific anaerobic fungi and the others representing the aerobic fungi. In addition, the unclassified *Opisthokonta* was the relative group of kingdom *Fungi* (Torruella et al., 2012).

Although these core taxa were consistently presented among almost all animals, we observed noticeable individual variations in their abundances (coefficient of variation ranged from 2.4 to 408.1%). Similar individual variations were reported in a recent study on active rumen prokaryotic microbiota (Li and Guan, 2017). Furthermore, consistent with our findings, Comtet-Marre et al. (2017) reported that the active rumen eukaryotic microbiota was dominated by ciliates (*Intramacronucleata*), flagellates (*Excavata*), and anaerobic fungi (*Neocallimastigomycota*). The authors investigated eukaryotic diversity in rumen samples obtained from a lactating dairy cow using metatranscriptomics, wherein the enriched V3–V4 regions of 18S rRNA were used for taxonomic annotation of eukaryotes. However, these studies did not try to address a potential commensal role of rumen flagellated protozoa or aerobic fungi, which was further explored in our study. This inconsistency might be due to the difference on the fraction of data being analyzed and on the focus of these studies. Earlier studies were mainly focused on rumen ciliates, and the taxonomic assignment was made based on sequence data generated using ciliate-specific marker gene (Kittelmann et al., 2013; Henderson et al., 2015; Kittelmann et al., 2015; Comtet-Marre et al., 2017). Although the rumen ciliate reference database used in these studies was based on a full-length 18S rRNA gene, it was built for the analysis of *Trichostomatia* and thus does not contain reference sequences of flagellated protozoa. Similar for rumen fungi, previous studies were all focused on anaerobic fungi. In our current study, the entirety of 18S rRNA gene sequences was extracted from the metatranscriptomic dataset and used for taxonomic analysis, which was more representative for overall rumen eukaryotic community. However, the metatranscriptome dataset-based taxonomic rumen eukaryota assessment may also be biased due to the depth of sequences, annotated 18S rRNA transcripts, and still limited information of full-length 18S rRNA genes in the SILVA database. To get better understanding of rumen eukaryotes, a combined approach is needed. It is necessary to generate and analyze the metatranscriptome data with SILVA first to understand the overall representation of all eukaryotes, and then the information can be used to discover marker genes for anaerobic fungi and ciliate protozoa in greater detail using the available fine-resolution frameworks.

### Compositional Profiles of the Active Rumen Protozoal Community

**FIGURE 2:**
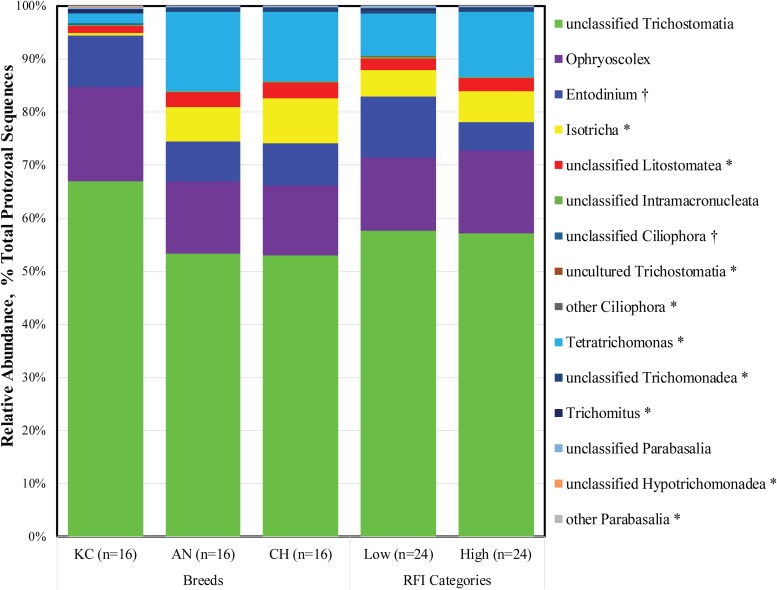
Active rumen protozoal composition at the genus level in beef cattle of three breeds and with divergent residual feed intake (RFI). Taxa name with asterisk (*) represents significant breed effect (*P* < 0.05), and taxa name with dagger (†) represents significant RFI effect (*P* < 0.05).

As described above, *Ciliophora* and *Parabasalia* were the top two active rumen protozoal phyla detected in this study. Further analysis revealed a total of 11 and 7 taxa at the genus level being identified within the *Ciliophora* and *Parabasalia* phylum, respectively, with eight and five taxa being detected, respectively (Figure 2). Within the phylum *Ciliophora*, the top five abundant genera were unclassified *Trichostomatia* (26.90 ± 1.09%), *Ophryoscolex* (6.89 ± 0.42%), *Entodinium* (3.90 ± 0.49%), *Isotricha* (3.72 ± 0.53%), and unclassified *Litostomatea* (1.12 ± 0.14%). Within the phylum *Parabasalia*, *Tetratrichomonas* (4.78 ± 1.26%), unclassified *Trichomonadea* (0.31 ± 0.07%), *Trichomitus* (0.12 ± 0.07%), and unclassified *Parabasalia* (0.10 ± 0.02%) were dominant and prevalent in all 48 samples (Supplementary Table S1).

As a phylum of ciliate protozoa, *Ciliophora* is distinguishable from other protozoa by a number of specialized features, including the procession of hair-like cilia, the presence of two types of nuclei at some stage in their life cycle, and a unique form of sexual reproduction called conjugation (Warren and Esteban, 2019). Ciliates are the most abundant protozoa found in the rumen of both domesticated and wild ruminants (Newbold et al., 2015), and they have been extensively studied as representatives of rumen protozoa because of their high prevalence (Newbold et al., 2015; Puniya et al., 2015). The present study further confirmed that ciliates were dominant in the active rumen eukaryotic community. The rumen ciliates were assigned based on the 18S rRNA sequence from the metatranscriptome dataset in this study, and 18S rRNA is the active center of protein synthesis in the 40S ribosomal subunit. Increased numbers of ribosomes, which lead to increased amounts of RNA transcription and protein synthesis, are presumed to be proportional to increases in 18S rRNA (Hayashi, 2014). Therefore, the high proportion of active rumen ciliates might suggest their importance in rumen metabolism.

To date, more than 250 species of ciliates belonging to 1 class (*Litostomatea*), 2 orders (*Vestibuliferida* and *Entodiniomorphida*), 16 families, and at least 25 genera have been identified in the forestomach and large intestine of herbivorous animals (Kittelmann and Janssen, 2011; Puniya et al., 2015). The present study also revealed that 99.99% of the 18S rRNA sequences from the identified active rumen ciliates belonged to the class *Litostomatea*, although sequences belonging to class *Spirotrichea* were also identified in five animals. However, only three genera (*Ophryoscolex*, *Entodinium*, and *Isotricha*) were annotated, and 52.81% of the active ciliates were categorized as the unclassified *Trichostomatia*. These observations might ascribe to the employed reference database (Silva 18S rRNA gene sequences; release 132), which contains very few almost-full-length entries that can serve as references for deeper-level phylogenetic identification of sequencing reads (Kittelmann and Janssen, 2011). A recent survey based on 18S rRNA gene amplicon sequencing revealed that almost all protozoal sequencing data from 742 rumen content samples worldwide were assigned to 12 genus-equivalent protozoal groups, namely, *Anoplodinium-Diplodinium*, *Enoploplastron*, *Entodinium*, *Epidinium*, *Eremoplastron-Diploplastron*, *Eudiplodinium*, *Metadinium*, *Ophryoscolex*, *Ostracodinium*, *Polyplastron*, *Dasytricha*, and *Isotricha* (Henderson et al., 2015). Similar rumen ciliate community structure has also been observed in cattle using high-throughput sequencing and microscopic methods (Kittelmann and Janssen, 2011; Kittelmann et al., 2015). The differences between metatranscriptomic and traditional identification methods further emphasize that more isolated and pure culture-based studies are needed to comprehensively characterize rumen eukaryotic microorganisms.

The *Parabasalia* are a clade of single-celled, anaerobic, flagellated protozoa that have evolved as symbionts or parasites in the digestive tracts of insects and vertebrates. Much of the known parabasalians occur in the gut of termites and their sister lineage, where they contribute to wood digestion as part of a complex microbial community. Compared to ciliated protozoa, the flagellated protozoa have been largely neglected, with very little information available about them (Williams et al., 1992). The present study revealed that active flagellates accounted for 11.45% of the total identified rumen protozoa, although considerable individual variation was observed (from 0.02 to 93.60%). Meanwhile, four taxa of parabasalians at the genus level were observed in all samples, suggesting that they are prevalent in the rumen of beef cattle. In addition, flagellated protozoa of the dominant genus *Tetratrichomonas* were also detected in the bovine rumen using traditional microscopy (Williams et al., 1992). To our knowledge, this is the first study to report the high prevalence of the parabasalians in the rumen of beef cattle. Furthermore, majority of the described parabasalians are obligate symbionts present in the gut of wood-eating insects and help their host to digest cellulose, in cooperation with the other microorganisms present in the intestine (Čepička et al., 2016). Similarly, ruminants also feed on plant structural carbohydrates such as lignin, cellulose, and hemicellulose, which can be hydrolyzed by the symbiotic microbiota *via* producing highly active lignocellulolytic enzymes (Puniya et al., 2015). Therefore, it is speculated that the flagellated protozoa are derived from feed and play an important role in fiber digestion.

Notably, correlation analysis revealed that there was a negative correlation between the relative abundance of phylum *Parabasalia* and *Ciliophora* (*R*^2^ = –0.83, *P* < 0.0001). Previous studies have also shown that flagellates can be observed in the calf rumen in early life, but their numbers decrease when ciliated protozoa become established (Williams et al., 1992). These data suggest that both these two groups of protozoa may compete for existence in the rumen, although the mechanism is unclear. Considering the negative correlation between the two protozoal groups, along with the potential role of flagellates in the digestion of plant materials, adequate attention should be paid to the rumen flagellate community in future studies.

### Compositional Profiles of the Active Rumen Fungi Community

Rumen fungi are generally considered as anaerobic fungi because they inhabit the rumen and alimentary tract of mammalian herbivores, which are typically deoxygenated. These anaerobic fungi are categorized as a distinct phylum, *Neocallimastigomycota*, which is the earliest diverging lineage unequivocally assigned to the kingdom *Fungi* (Gruninger et al., 2014). The anaerobic fungi are considered to contribute significantly to the overall metabolism of their host by playing a major role in the degradation of lignified plant tissues (Gruninger et al., 2014). Interestingly, in the present study, the phylum *Neocallimastigomycota* only accounted for 43.47% of the total fungal sequences, suggesting that the active rumen fungal community is more diverse than previously appreciated. Meanwhile, substantial amounts of RSVs were classified as obligate aerobes belonging to the phyla *Basidiomycota, Mucoromycota*, *Ascomycota*, *Chytridiomycota, Mucoromycota*, and unclassified *Fungi*. Although how these aerobic microorganisms survive in the rumen remains unclear, it is speculated that they play a major role in scavenging oxygen entering the rumen. This might have beneficial effects on the anaerobic fermentation process for other obligately anaerobic microbes in the rumen.

**FIGURE 3:**
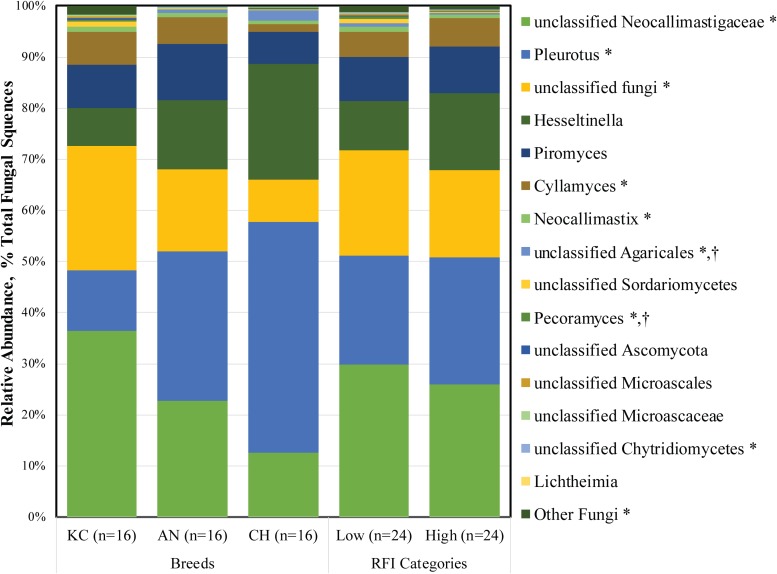
Active rumen fungal composition at the genus level in beef cattle of three breeds and divergent residual feed intake (RFI). Taxa name with asterisk (*) represents significant breed effect (*P* < 0.05), and taxa name with dagger (†) represents significant RFI effect (*P* < 0.05).

At the genus level, a total of 26 fungal taxa were identified, of which 15 were considered as detected (Figure 3). Among them, five taxa belonged to the phylum *Neocallimastigomycota*, while others belonged to the aerobic fungal taxa. Unclassified *Neocallimastigaceae* (1.30 ± 0.31%), *Piromyces* (0.40 ± 0.07%), *Cyllamyces* (0.24 ± 0.06%), *Neocallimastix* (0.04 ± 0.01%), *Pleurotus* (1.04 ± 0.09%), unclassified *Fungi* (0.88 ± 0.20%), and *Hesseltinella* (0.55 ± 0.03%) were the top seven active taxa in the rumen fungal community (Supplementary Table S1). To date, 10 genera, including monocentric *Piromyces*, *Neocallimastix*, *Caecomyces*, *Buwchfawromyces*, *Oontomyces*, *Pecoramyces*, *Feramyces*, and polycentric *Cyllamyces*, *Orpinomyces*, and *Anaeromyces*, have been described under the phylum *Neocallimastigomycota* according to culture-based and culture-independent approaches (Gruninger et al., 2014; Hanafy et al., 2017, 2018a,b). Among them, four genera, including *Piromyces*, *Neocallimastix*, *Cyllamyces*, and *Pecoramyces*, along with unclassified *Neocallimastigaceae*, were identified as the active fungal taxa present in at least 32 of the total 48 samples in this study.

As the most prevalent rumen aerobic fungi, the genus *Pleurotus* and *Hesseltinella* belonging to the phylum *Basidiomycota* and *Mucoromycota* accounted for 22.66 and 11.91% of the total active rumen fungal sequences, respectively. Although, to the best of our knowledge, this is the first study in which active aerobic fungi were observed in the rumen, we believed that these fungi are true rumen colonizers. First, these fungi were identified from metatranscriptomic rather than metagenomic datasets, suggesting that they are live microorganisms in the rumen rather than dead or inactive microbes from the diets. Second, the prevalence and substantial proportion of *Pleurotus* (1.04%) and *Hesseltinella* (0.55%) in the active rumen eukaryotic microbiome suggested that these fungal sequences were not derived from contingent or chimera sequences generated from RNA-seq. Third, the rumen is not an obligate anaerobic environment as some amounts of oxygen enter the rumen along with the feed (Santra and Karim, 2003), facilitating the survival of these aerobic fungi. Additionally, the genus *Pleurotus* is one of the most efficient white rot fungi lignocellulose degrader and is generally cultivated on non-composted lignocellulosic substrates (Bellettini et al., 2019). The *Hesseltinella* is a genus of fungi under the order *Mucorales*, comprising predominantly saprotrophs inhabiting soil, drug, and dead plant material, as well as several parasites on plants (Walther et al., 2013). Thus, these fungi could be ingested by the ruminants along with the plant material, thereby colonizing in the rumen. Moreover, the genus *Pleurotus* has exceptional ligninolytic properties by which it cleavages cellulose, hemicellulose, and lignin from wood (Machado et al., 2016). Mucoralean strains have been used for centuries in the fermentation of traditional Asian and African food (Nout and Aidoo, 2010), and they also play a role in the production of various kinds of cheese. Therefore, it is expected that the presence of active *Pleurotus* and *Hesseltinella* species in the rumen might contribute to the degradation of dietary fiber, although their role in the rumen is currently unknown. Considering their potential role in the degradation of lignocellulosic plant fiber and scavenging of oxygen that enters the rumen, the functionality and metabolism of these aerobic fungi warrant further study.

### Effect of Breed on the Rumen Eukaryotic Microbiome

Phenotypic data, including RFI, ADG, and DMI, were obtained from a study by Li et al. (2019a) and were reanalyzed to compare the overall difference among three breeds using the mixed effect model as described in the section “Statistical Analysis.” Results showed that no interaction effect between breed and feed efficiency (*P* > 0.05) was observed. The RFI values were not affected by breed (*P* = 0.12). However, KC steers had a lower DMI (*P* < 0.05) than the other two breeds. Meanwhile, AN steers had a higher ADG (*P* < 0.05) than KC steers, but the ADG of both these breeds was not significantly different (*P* > 0.05) from that of CH steers. To comprehensively investigate the effects of breed and RFI on rumen eukaryotic diversity, we treated the eukaryotic microbiota as a whole to combine both protozoal and fungal datasets. The rarefaction curves based on the Shannon index (Supplementary Figure S1A) and Faith’s PD (Supplementary Figure S1B) for each sample appeared to level out as the sampling depth outnumbered 300,000, indicating that the lowest sequence count of the samples (549,095) in this study was sufficient to assess ruminal eukaryotic diversity.

**FIGURE 4:**
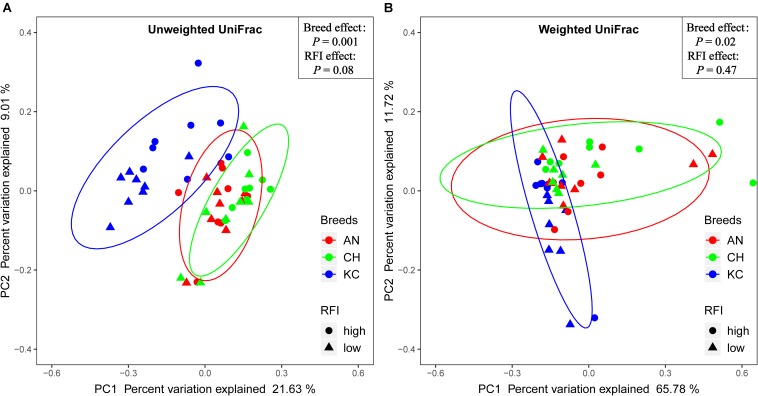
Similarity of the rumen eukaryotic profiles between beef cattle with divergent residual feed intake (RFI) from three breeds. Distance between samples based on similarity in representative sequence variants (RSVs) composition was calculated using unweighted UniFrac **(A)** and weighted UniFrac distance **(B)** and visualized using principal coordinate analysis (PCoA) plots. The impact of breed and RFI on the clustering pattern of microbial communities was tested using PERMANOVA. The AN, CH, and KC represent Angus, Charolais, and Kinsella Composite beef steers, respectively. The ovals in blue, red, and green represent 95% confidence interval of KC, AN, and CH, respectively.

Venn diagram analysis showed that a total of 5,015 RSVs were commonly presented in cattle rumen of all the three breeds, and 15,907, 7,096, and 5,001 RSVs were uniquely identified in cattle rumen of KC, AN, and CH steers, respectively (Supplementary Figure S2A). Further analysis showed that breed had no effect on the alpha-diversity indices (*P* > 0.05) (Table 1), suggesting that there was no significant difference in active eukaryotic richness among the three breeds. However, PCoA based on unweighted UniFrac distance showed that these samples were clustered by breed (PERMANOVA; *P* = 0.001; Figure 4A), indicating that the phylogenetic lineages of rumen eukaryotes among the three breeds were significantly distinct from each other. However, this distinctiveness was reduced when taking the relative abundance of RSVs into consideration, as demonstrated by weighted UniFrac distance PCoA plots (PERMANOVA; *P* = 0.02; Figure 4B), where only active rumen eukaryotic profiles of KC were separated from those of the other two breeds (PERMANOVA; *P* = 0.0015). This result was expected because unweighted UniFrac distance only considers species presence/absence information in a microbial community, while weighted UniFrac incorporates RSVs abundance information (Chen et al., 2012), and thus the high proportion of the overlapped unclassified eukaryotes across all samples decreases the power over weighted UniFrac distance for detecting the differences among microbial communities (Chen et al., 2012). Hence, our results suggest that breed has no effect on species richness but significantly affects the PD of the rumen eukaryotic community.

**TABLE 1:** Effects of breed and RFI on the alpha diversity indices of rumen eukaryotic communities.

**Items**	**Breed^1^**			**RFI^2^**		**SEM**	***P*-value**		
	**AN**	**CH**	**KC**	**High**	**Low**		**Breed**	**RFI**	**Breed × RFI**
Pielou’s evenness	0.78	0.79	0.76	0.77	0.78	0.01	0.09	0.32	0.65
Faith’s PD	137	126	143	124^b^	147^a^	5	0.24	<0.01	0.06
Observed OTUs	1,937	2,106	2,287	1,921^b^	2,299^a^	73	0.11	<0.01	0.21
Shannon index	8.60	8.58	8.49	8.40^b^	8.72^a^	0.07	0.75	0.02	0.37

*^1^AN, CH, and KC represent Angus, Charolais, and Kinsella Composite steers, respectively. ^2^OTUs, operational taxonomic units; PD, phylogenetic diversity; RFI, residual feed intake; values within each row with different superscripts are different.*

**FIGURE 5:**
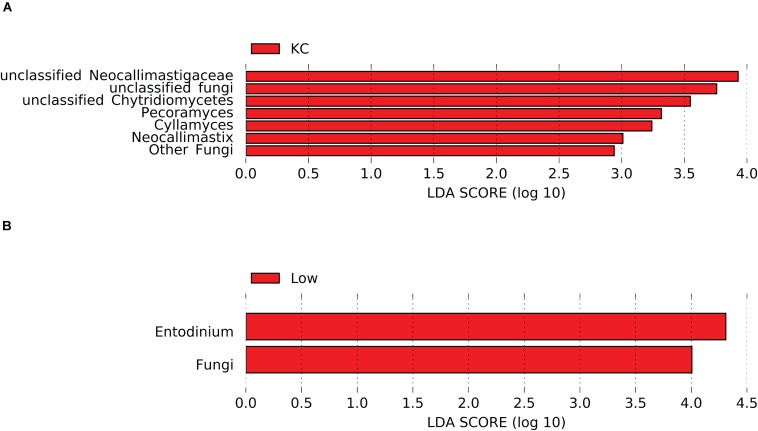
Differential rumen eukaryotic features between the three beef cattle breeds **(A)** and high- and low-residual feed intake (RFI) steers **(B)** by linear discriminant analysis effect size (LEfSe). KC, Kinsella Composite beef steers; Low, steers with low RFI.

Differential abundance analysis showed that the relative abundance of 26 eukaryotic taxa was affected by breed (*P* < 0.05, Supplementary Table S1). Considering that most of the active eukaryotic taxa were not affected by the interaction between breed and RFI (*P* > 0.05, Supplementary Table S1), a more stringent LEfSe analysis was performed to identify differentially abundant taxa among the three breeds. Results revealed that seven taxa detected at the genus level were affected by breed (LDA score > 2 and *P* < 0.05), with KC steers exhibiting high abundance of unclassified *Neocallimastigaceae*, *Pecoramyces*, and *Cyllamyces*, etc. (Figure 5A). Noticeably, all these seven taxa belonged to the rumen fungi, suggesting that the fungal community was more active in the rumen of KC steers than that in the rumen of AN and CH steers.

Similar host genetic background effects on gut microbial profiles have been observed in recent studies based on amplicon sequencing, in which samples from various mammals were investigated (Henderson et al., 2015; Paz et al., 2016; Groussin et al., 2017). Furthermore, a recent metatranscriptome-based study also revealed that breed did not influence the richness of active bacterial and archaeal communities, while the active rumen prokaryotic communities in KC were distinct from those in AN and CH steers (Li et al., 2019a) from the same metatranscriptomic datasets. These studies suggest that host genetic background can influence the activity of the entire rumen microbiota, including both prokaryotes and eukaryotes. Nevertheless, our knowledge on the role of host in regulating microbiota is currently limited. Several breed-associated biological factors, such as eating frequency, DMI, and rumen size potentially contribute to the rumen microbiota variations observed among various breeds, as discussed by Li et al. (2019a). However, the mechanisms by which these factors influence the microbial community have not been well described. Therefore, further studies to link these biological factors to eukaryotic compositional profiles are needed to better understand the effect of breed on rumen microbiota.

### Effect of Residual Feed Intake on the Rumen Eukaryotic Microbiome

Phenotypic data, including RFI, ADG, and DMI were obtained from a study by Li et al. (2019a) and were reanalyzed to compare the overall difference between high- and low-RFI categories, as mentioned above. Results showed that the RFI values were significantly different between high- and low-RFI steers (0.86 vs. –0.78 kg/day; *P* < 0.0001), and high-RFI animals had a higher DMI than low-RFI individuals (10.73 vs. 9.55 kg/day; *P* < 0.0001). Venn diagram analysis showed that a total of 10,808 RSVs were commonly presented in cattle rumen of all RFI groups, and 16,598 and 10,658 RSVs were uniquely identified in rumen of low- and high-RFI steers, respectively (Supplementary Figure S2B). Moreover, majority of the alpha diversity indices were affected by RFI, with Faith’s PD, observed OTUs, and Shannon index in low-RFI steers being higher (*P* < 0.05) than those in high-RFI steers (Table 1). These results suggest that low-RFI steers exhibit higher eukaryotic richness in the rumen compared with high-RFI steers. However, the PCoA did not show a clear separation of the active rumen eukaryotic community between high- and low-RFI steers based on the unweighted and weighted UniFrac matrices of RSVs (Figure 4). In addition, PERMANOVA did not test the statistical differences (*P* > 0.05) in the eukaryotic communities between high- and low-RFI steers, although a trend (*P* = 0.08) of difference was observed between these two groups based on the unweighted UniFrac distance.

Differential abundance analysis showed that the relative abundance of seven eukaryotic taxa was affected by RFI (*P* < 0.05, Supplementary Table S1). The more stringent LEfSe analysis further confirmed that the relative abundance of two eukaryotic taxa (kingdom *Fungi* and genus *Entodinium*) was significantly different (LDA score > 2 and *P* < 0.05) between steers with divergent RFI (Figure 5B). Low-RFI steers had an approximately twofold higher abundance of active kingdom *Fungi* and genus *Entodinium* (5.52 ± 1.03 and 5.23 ± 0.74%, respectively) in the rumen than high-RFI steers (3.66 ± 0.84 and 2.58 ± 0.53%, respectively). As discussed above, most of the detected rumen fungal taxa had the ability to degrade the recalcitrant lignocellulosic components of the feed particles by producing all the enzymes necessary for plant material decomposition, including cellulolytic and hemicellulolytic enzymes. These hydrolases enable the rumen fungi to penetrate the plant cell walls, access fermentable substrates not available to surface-acting bacteria, colonize the sturdy plant structures, and weaken and degrade the plant tissues, thus reducing the size of plant particles (Puniya et al., 2015). Meanwhile, the invasive rhizoidal growth of rumen fungi possibly aids substrate decomposition mechanically (Gruninger et al., 2014). Enzymatic and mechanical degradation of plant material by anaerobic fungi provides an increased surface area for bacterial colonization, resulting in an increase in the degradation of plant cell wall (Gruninger et al., 2014). Therefore, the high abundance of active rumen fungi in low-RFI steers may provide increased energy to hosts from the fermentation of structural polysaccharides, leading to a high feed efficiency. As an indirect proof, it has been demonstrated that inclusion of cultures of anaerobic fungi in the diets of various ruminants improved feed intake, animal growth rate, feed efficiency, and milk production (Lee et al., 2000; Saxena et al., 2010; Paul et al., 2011).

Additionally, a high abundance of *Entodinium* was detected in the rumen of low-RFI animals. Inconsistent with the findings of our study, Carberry et al. (2012) reported that the RFI phenotype had no effect on the relative abundance of genus *Entodinium*, as indicated by qPCR analysis. This inconsistency might be due to the differences in genetic material employed (DNA vs. RNA) and targeted microbial populations (several microbial taxa vs. entire active eukaryotic microbiome). Members belonging to the genus *Entodinium* are the smallest, simplest, and most common rumen ciliates and are found in almost all ruminants (Williams et al., 1992). *Entodinium* species are known starch degraders, and starch is essential for their maintenance and growth (Williams et al., 1992). A recent microscopy-based study found that the proportion of *Entodinium* in the total rumen protozoal community could reach 93% in the rumen of beef cattle fed a high concentrate-based diet (Yuste et al., 2019). The starch engulfed by *Entodinium* species was fermented into acetic acid and butyric acids as the major end products (together 80–90% of the total volatile fatty acids), accompanied by the production of lesser amounts of formic acid and propionic acid and some carbon dioxide and hydrogen (Abmu Akkada and Howard, 1960). Furthermore, it was shown that ruminal starch digestibility was increased from 84 (in the absence of protozoa) to 89% (in the presence of *Entodinium* species) (Veira et al., 1983). In the present study, the finishing diet consisted mainly of barley grain and barley silage, which were rich in starch. Therefore, the increased abundance of active *Entodinium* may provide more energy to hosts from the fermentation of starch, resulting in a high feed efficiency. On the other hand, *Entodinium* species have a substantial potential to engulf and degrade bacteria (Belanche et al., 2012) and metabolize lactic acid (Williams et al., 1992). Although the engulfment of bacteria by *Entodinium* species has been proved to be responsible for much of the bacterial protein turnover (Newbold et al., 2015), the loss of bacterial nitrogen due to protozoal activity can be compensated by the increased digestibility of protozoal bodies (Williams et al., 1992). Furthermore, the protozoal fermentation of the ingested starch is less rapid, and a detrimental volatile fatty acid buildup does not occur (Williams et al., 1992). Therefore, a higher abundance of *Entodinium* species may have a significant stabilizing effect on ruminal fermentation and might play an important role in regulating ruminal lactate metabolism and preventing the occurrence of lactic acid acidosis. This is important to animals receiving high-energy diets and contributes to the high feed efficiency of the steers in the current study.

## Conclusion

In the current study, we investigated the compositional characteristics of active rumen eukaryotic microbiome using metatranscriptomics and revealed a core active eukaryotic community in the rumen of beef steers. Taxonomic analysis revealed that the rumen eukaryotic community was more diverse than previously reported, and the identification of flagellated protozoa and aerobic fungi suggested that these previously unreported eukaryotic microbes could also play a role in rumen function. However, our results also highlight the need to characterize more eukaryotic genomes from rumen to further dissect their exact roles in the rumen microbiome. Further analysis revealed distinguishable rumen eukaryotic microbiota among the three beef cattle breeds. These breed-associated differences represent potential superiorities of each breed, which could further be applied to manipulate the rumen microbiome through selective breeding of the cattle. Moreover, two differentially abundant microbial taxa, the active protozoal genus *Entodinium* and the kingdom *Fungi*, were identified between steers with divergent RFIs, suggesting that these eukaryotes may contribute to variations in the feed efficiency of beef cattle. Overall, these findings extend our understanding on rumen eukaryotic communities and highlight the possibility of improving feed efficiency through altering rumen eukaryotic microbiota.

## Data Availability Statement

The datasets generated for this study can be found in the Sequence Read Archive (SRA) of National Center for Biotechnology Information (NCBI) under accession number PRJNA448333.

## Ethics Statement

The animal study was reviewed and approved by the Livestock Care Committee of the University of Alberta (No. AUP00000882).

## Author Contributions

YZ, FL, QM, and LG designed this study. FL and YC performed the RNA isolation and sequencing of library construction. YZ, FL, and LG conducted the bioinformatics and statistical analyses and data interpretation. YZ, QM, HW, and LG were responsible for the manuscript writing. All authors read and approved the final manuscript.

## Conflict of Interest

The authors declare that the research was conducted in the absence of any commercial or financial relationships that could be construed as a potential conflict of interest.
