# Supplementary material for: Metatranscriptomic Profiling Reveals the Effect of Breed on Active Rumen Eukaryotic Composition in Beef Cattle With Varied Feed Efficiency
Source: Front Microbiol. 2020 Mar 13;11:367. doi: 10.3389/fmicb.2020.00367 (PMC7082318; doi:10.3389/fmicb.2020.00367)
Supplement: Supplementary file 1 [file Data_Sheet_1.DOCX]

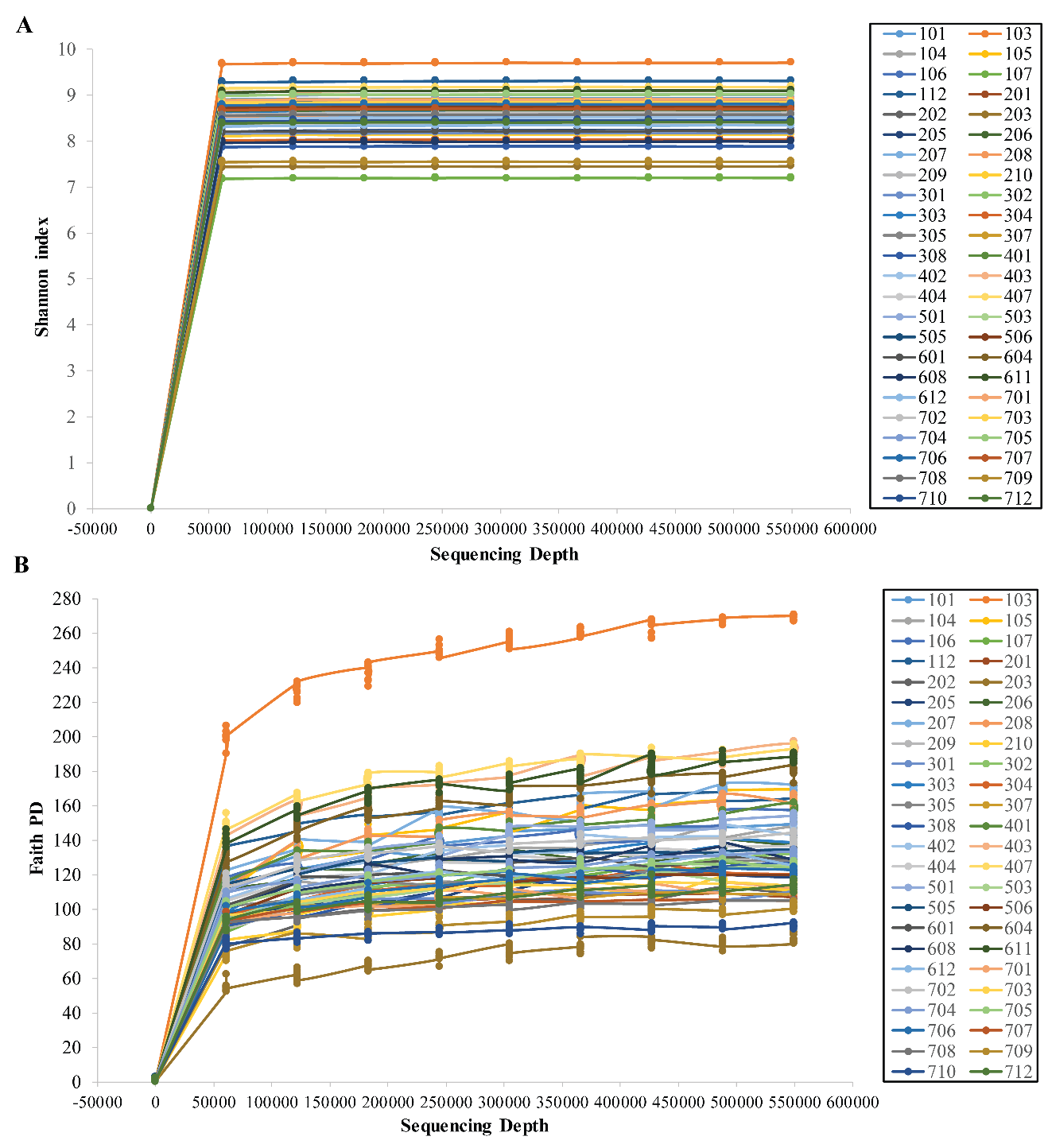


Figure S1. The alpha rarefaction plots based on Shannon index (A) and Faith PD (B) for each sample.


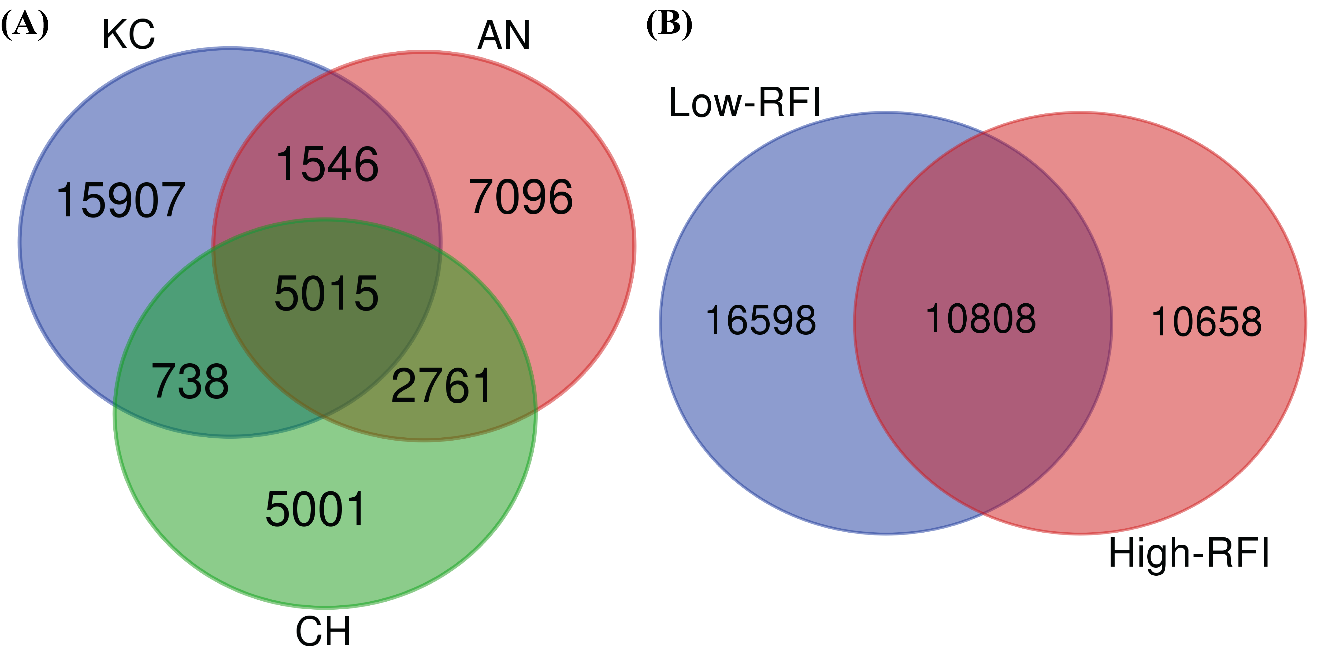


Figure S2. The RSVs that commonly or uniquely presented in cattle rumen of the three breeds (A) and divergent RFI groups (B) revealed by Venn diagram analysis.
